# Supplementary material for: Expression strategies for the efficient synthesis of antimicrobial peptides in plastids
Source: Nat Commun. 2022 Oct 4;13:5856. doi: 10.1038/s41467-022-33516-1 (PMC9532397; doi:10.1038/s41467-022-33516-1)
Supplement: Supplementary file 1 — Supplementary Information [file 41467_2022_33516_MOESM1_ESM.pdf]

Expression strategies for the efficient synthesis of antimicrobial peptides in plastids

Hoelscher *et al.*

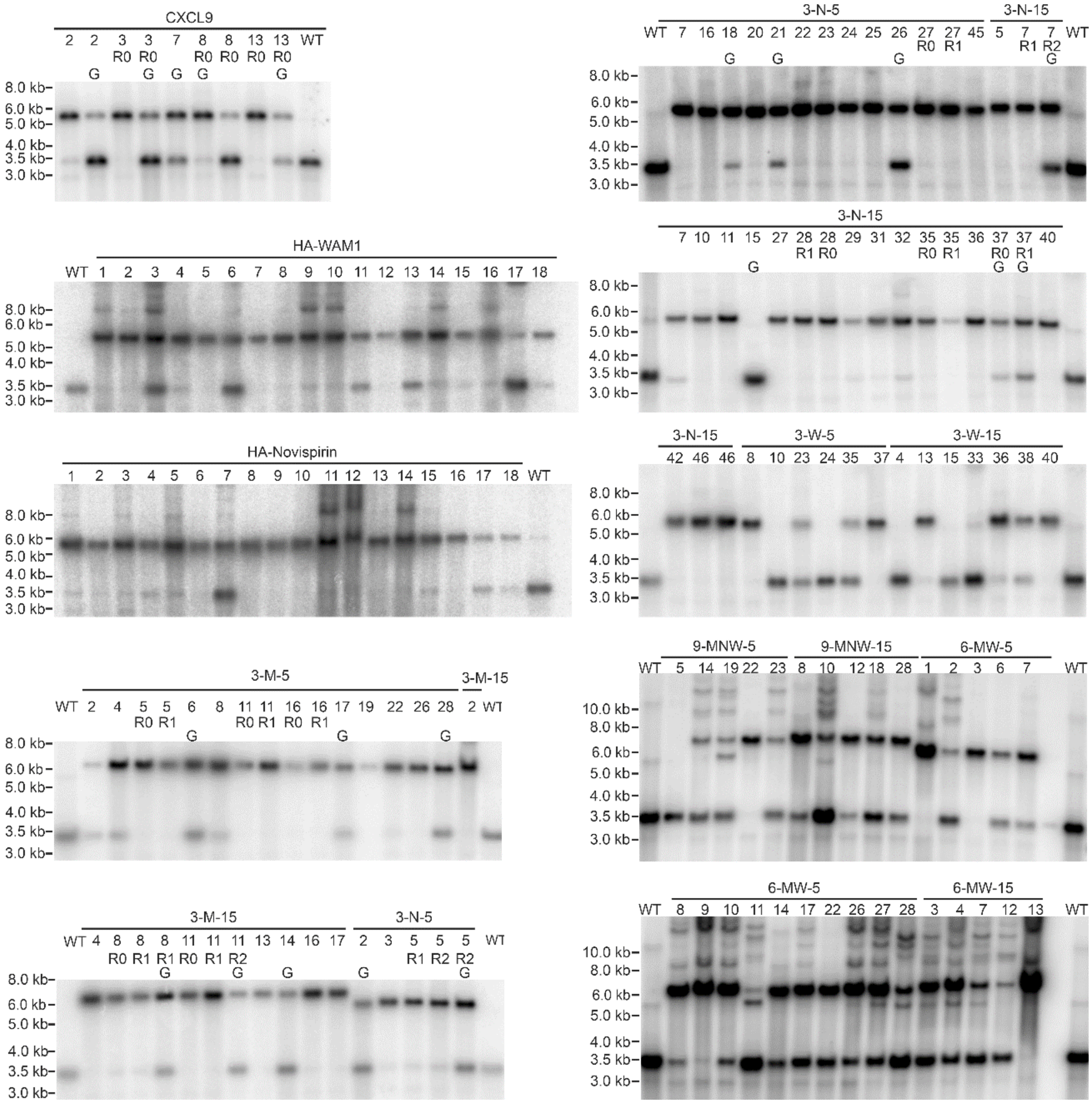

Supplementary Fig. 1. Identification of homoplasmic transplastomic Prrn::(f)AMP plants by Southern blotting. For verification of transgene integration by restriction fragment length polymorphism (RFLP) analysis, isolated total leaf DNA was digested with BglII, separated by agarose gel electrophoresis and blotted. This analysis produced a 3.5 kb restriction fragment for the wild type (WT), and a larger fragment for the transplastomic lines. The size of the expected restriction fragments for the transplastomic lines is at least 2 kb larger (due to foreign DNA shared by all constructs) and gains additional size by the different (f)AMP-encoding regions (Fig. 1; Supplementary Data 2). For some lines, different regeneration rounds were tested (R0: primary regeneration, R1: first regeneration, R2: second regeneration round). Samples from leaves that stood out by being green or partially green among a majority of pale regenerating plants are indicated with G. Numbers denote independent transformation events. For abbreviations, construct maps and information on the hybridization probe, see Fig. 1. These blots were performed once.

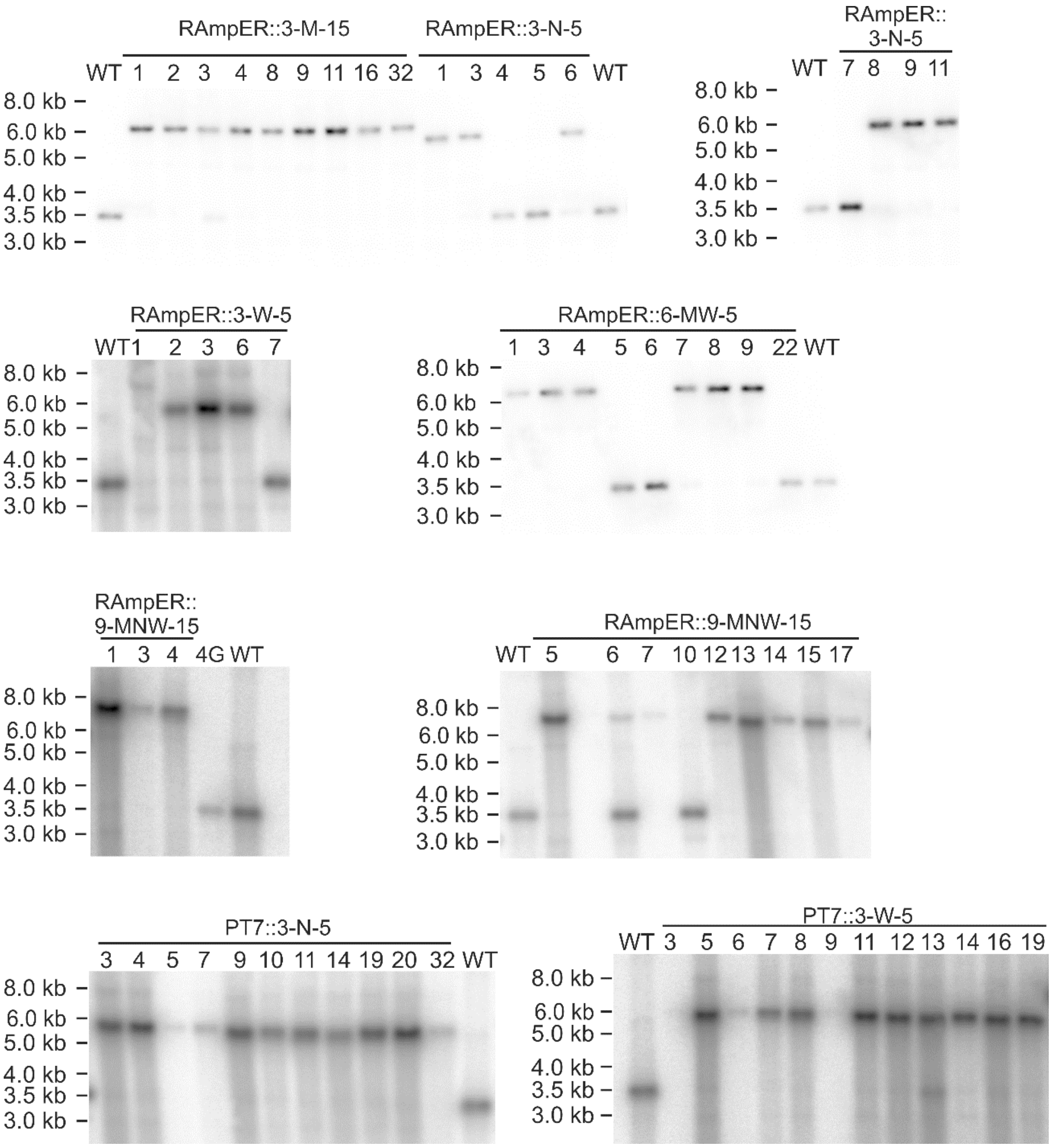

Supplementary Fig. 2. Southern blot analyses of transplastomic RAmpER and PT7 plants. For identification of homoplasmic plants, isolated DNA was digested with BglII and used for RFLP analysis. Hybridization is expected to detect a 3.5 kb fragment in the wild type and an at least 1.9 kb larger fragment in transplastomic plants, which gains additional size by the DNA encoding the different fAMPs (Fig. 3; Supplementary Data 2). Numbers denote independent transformation events. For abbreviations, construct maps and information on the hybridization probe, see Fig. 3. G: leaf sample that was (partially) green instead of pale green. These blots were performed once.

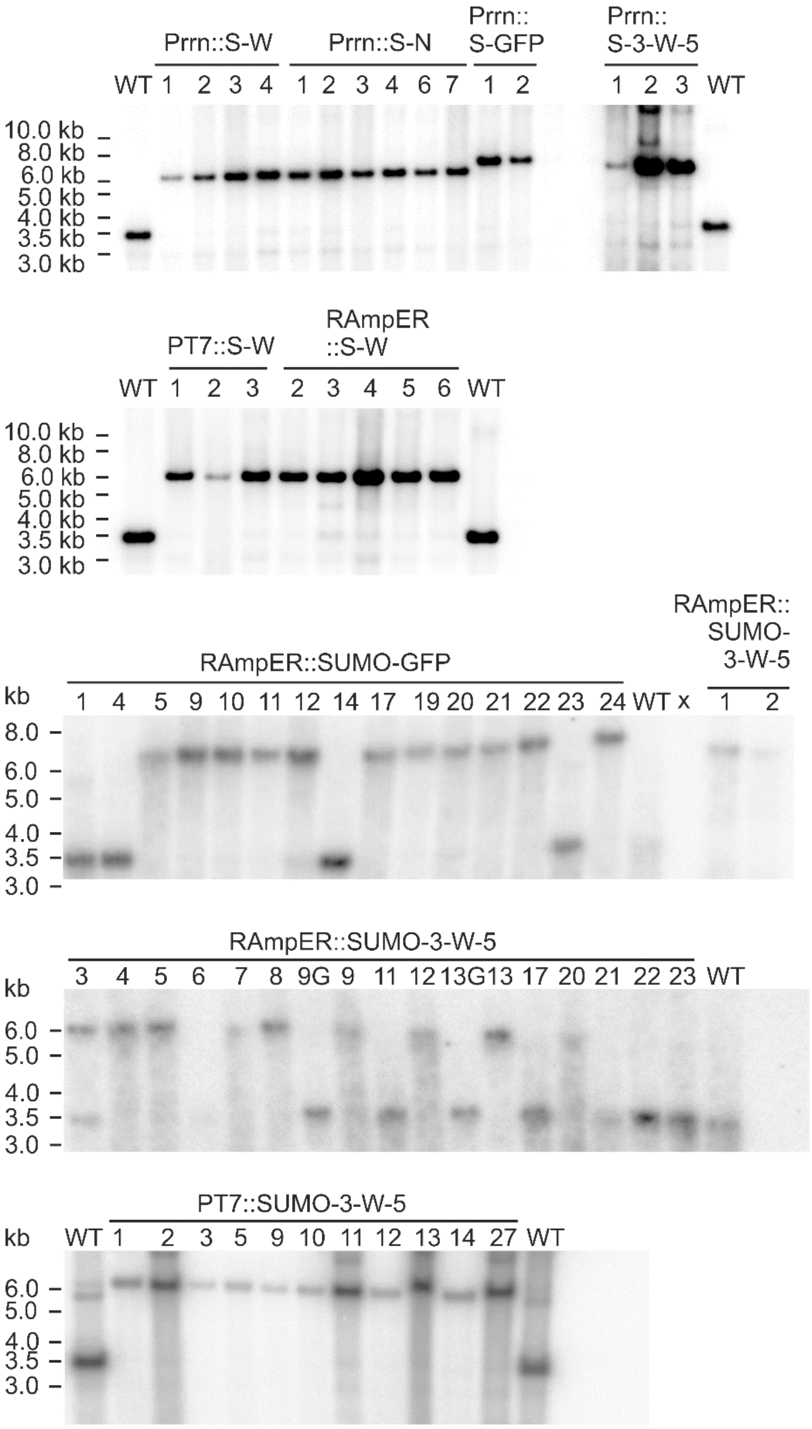

Supplementary Fig. 3. Identification of homoplasmic transplastomic plants expressing SUMO fusions by RFLP analysis. Isolated DNA was digested with BglII. For the wild type, Southern blot hybridization results in detection of a fragment of approximately 3.5 kb. In the transplastomic lines, the fragment sizes increase by the presence of shared elements (of 2.1 kb for PT7 constructs and 2.3 kb for Prrn constructs) plus the sizes of the inserted sequences encoding SUMO fusion proteins (Fig. 5; Supplementary Data 2). Numbers denote independent transformation events. For abbreviations, construct maps and information on the hybridization probe, see Fig. 5. These blots were performed once.

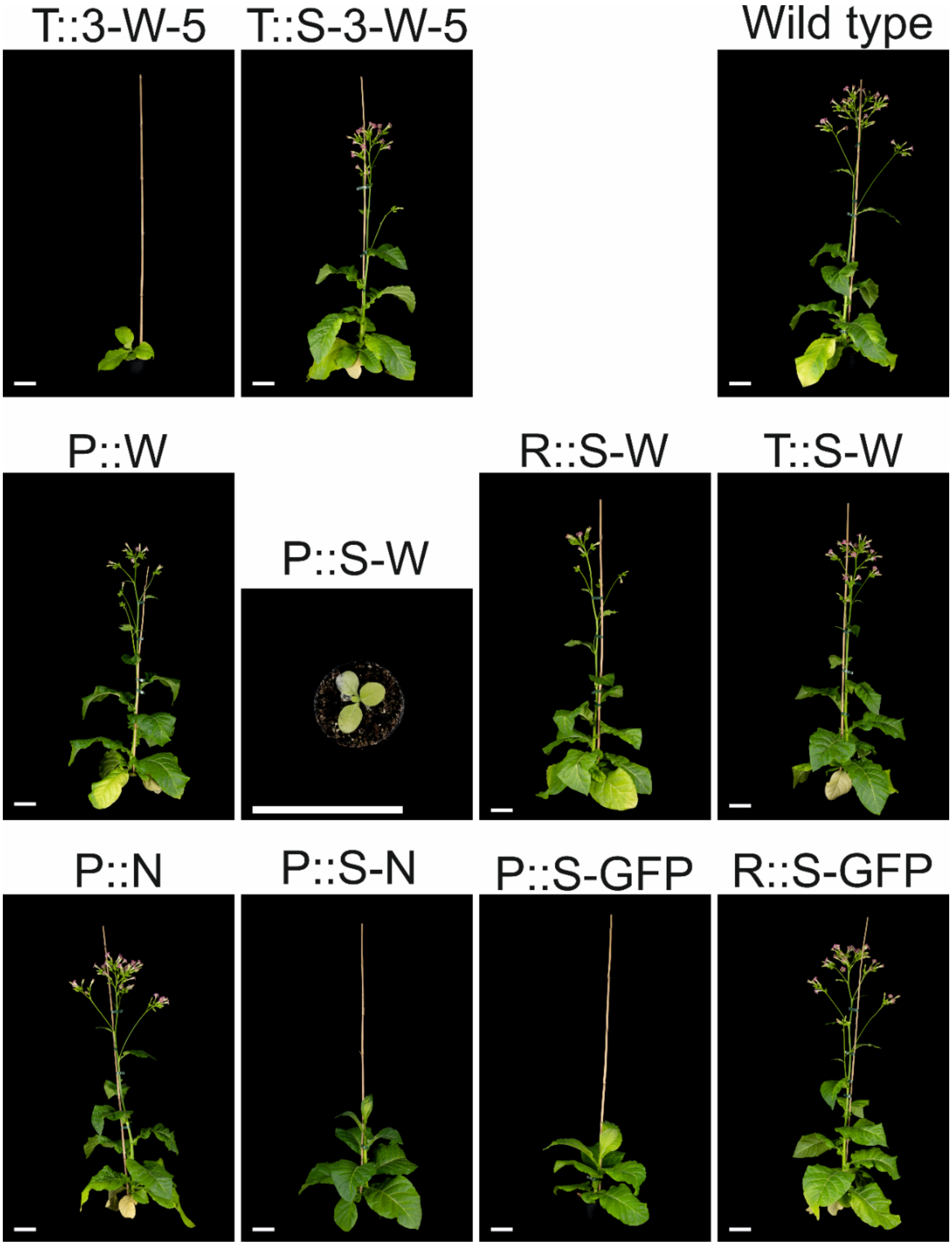

Supplementary Fig. 4. Effects of fusion to SUMO on plant phenotypes (two months after sowing). Plants expressing constructs with and without SUMO (S) and with different promoter systems (Prrn (P), RAmpER (R) & PT7 (T)) are compared. SUMO fusions were generated for 3-Wamdeposin-5 (3-W-5), HA-WAM1 (W), HA-Novispirin (N), and green fluorescent protein (GFP). Photos were taken two months after sowing. Scale bars: 10 cm.

Supplementary Table 1. DNA and amino acid sequences of the linkers used for the construction of fusion proteins. Lowercase letters indicate nucleotides leading to a less preferred codon that had to be introduced in order to reduce sequence similarity between the linkers.

Linker	Nucleotide sequences (5’ → 3’)	Amino acid sequence
L1	GGTGGTTCTGGTGGA	GGSGG
L2	GGAGGATCAGGTGGA	GGSGG
L3	GGTGGATCTGGTGGA	GGSGG
L4	GGTGGTAGTGGAGGT	GGSGG
L5	GGTGGTTCAGGTGGA	GGSGG
L6	GGAGGTAGTGGTGGT	GGSGG
L7	GGAGGTTCTGGAGGT	GGSGG
L8	GGTGGAAGTGGAGGT	GGSGG
L9	GGAGGTTCAGGAGGT	GGSGG
L10	GGTGGATCAGGAGGA	GGSGG
L11	GGTGGATCTTCATCATCTGGTGGTTCTTCTTCATCAGGTAGTGGA	GGSSSSGGSSSSGSG
L12	GGgGGTTCAAGTGGAGGgGGATCAGGAGGATCTTCTAGTTCAGGA	GGSSGGGSGGSSSSG
L13	GGTGGTGGATCATCAGGTTCTAGTTCTAGTGGAGGTGGTTCTGGT	GGGSSGSSSSGGGSG
L14	GGAGGAGGTAGTGGTTCAGGATCAAGTTCTTCAAGTGGTAGTGGA	GGGSGSGSSSSSGSG
L15	GGAGGAGGTTCcGGAGGAGGTGGATCcGGTGGATCTGGAAGTGGA	GGGSGGGGSGGSGSG
L16	GGTTCTTCTGGATCATCTAGTGGgGGTGGTGGTAGTTCTGGTGGA	GSSGSSSGGGGSSGG
L17	GGTTCATCTAGTTCTGGAAGTAGTGGTGGAGGTTCATCTTCAGGA	GSSSSGSSGGGSSSG
L18	GGTGGATCAGGATCTGGAGGTGGAGGATCATCATCAAGTGGTGGg	GGSGSGGGGSSSSGG
L19	GGAGGgGGgGGAGGAAGTGGAAGTTCAAGTTCAGGAGGTTCAGGA	GGGGGSGSSSSGGSG
L20	GGTAGTTCTTCATCTGGTAGTAGTGGATCTAGTTCATCTGGAGGA	GSSSSGSSGSSSSGG

Supplementary Table 2. Overview of possible (f)AMP and promoter system combinations. Combinations generated in this work are marked with X.

Polypeptide name	Expression system in chloroplasts		
	Prrn	RAmpER	PT7
HA-Novispirin	X		
SUMO-HA-Novispirin	X		
HA-WAM1	X		
SUMO-HA-WAM1	X	X	X
CXCL9	X		
3-Molluxubin-5	X		
3-Molluxubin-15	X	X	
3-Novescandin-5	X	X	X
3-Novescandin-15	X		
3-Wamdeposin-5	X	X	X
3-Wamdeposin-15	X		
SUMO-3-Wamdeposin-5	X	X	X
6-MW-5	X	X	
6-MW-15	X		
9-MNW-5	X		
9-MNW-15	X	X	
SUMO-GFP	X	X	
