# Supplementary material for: Expression strategies for the efficient synthesis of antimicrobial peptides in plastids
Source: Nat Commun. 2022 Oct 4;13:5856. doi: 10.1038/s41467-022-33516-1 (PMC9532397; doi:10.1038/s41467-022-33516-1)
Supplement: Supplementary file 3 — Description of Additional Supplementary Files [file 41467_2022_33516_MOESM3_ESM.pdf]

Description of Additional Supplementary Files

File Name: Supplementary Data 1
Description: List of AMPs that were selected for recombinant production in chloroplasts, their biological activities and physicochemical properties, proposed mode of action, amino acid sequence, source organism, peptide family and corresponding literature references (Supplementary references).

File Name: Supplementary Data 2
Description: Description of DNA constructs for chloroplast transformation

File Name: Supplementary Data 3
Description: Recombinant protein yield quantification, yield calculation excluding SUMO and SUMO cleavage quantification.
