# Supplementary material for: Expression strategies for the efficient synthesis of antimicrobial peptides in plastids
Source: Nat Commun. 2022 Oct 4;13:5856. doi: 10.1038/s41467-022-33516-1 (PMC9532397; doi:10.1038/s41467-022-33516-1)
Supplement: Supplementary file 7 — Reporting Summary [file 41467_2022_33516_MOESM7_ESM.pdf]

nature portfolio

Corresponding author(s): Ralph Bock

Last updated by author(s): Sep 19, 2022

Reporting Summary

Nature Portfolio wishes to improve the reproducibility of the work that we publish. This form provides structure for consistency and transparency in reporting. For further information on Nature Portfolio policies, see our Editorial Policies and the Editorial Policy Checklist.

Statistics

For all statistical analyses, confirm that the following items are present in the figure legend, table legend, main text, or Methods section.

n/a	Confirmed	
☒	☐	The exact sample size (*n*) for each experimental group/condition, given as a discrete number and unit of measurement
☒	☐	A statement on whether measurements were taken from distinct samples or whether the same sample was measured repeatedly
☒	☐	The statistical test(s) used AND whether they are one- or two-sided<br>*Only common tests should be described solely by name; describe more complex techniques in the Methods section.*
☒	☐	A description of all covariates tested
☒	☐	A description of any assumptions or corrections, such as tests of normality and adjustment for multiple comparisons
☒	☐	A full description of the statistical parameters including central tendency (e.g. means) or other basic estimates (e.g. regression coefficient) AND variation (e.g. standard deviation) or associated estimates of uncertainty (e.g. confidence intervals)
☒	☐	For null hypothesis testing, the test statistic (e.g. *F*, *t*, *r*) with confidence intervals, effect sizes, degrees of freedom and *P* value noted<br>*Give P values as exact values whenever suitable.*
☒	☐	For Bayesian analysis, information on the choice of priors and Markov chain Monte Carlo settings
☒	☐	For hierarchical and complex designs, identification of the appropriate level for tests and full reporting of outcomes
☒	☐	Estimates of effect sizes (e.g. Cohen's *d*, Pearson's *r*), indicating how they were calculated

Our web collection on statistics for biologists contains articles on many of the points above.

Software and code

Policy information about availability of computer code

Data collection	No code was used to collect the data in this study.
Data analysis	Calculation of protein concentration based on spectrophotometric data from BCA assay was done with Microsoft Excel (Microsoft 365 version 2202).<br>Cleavage efficiency of SUMO protease was determined by image quantification of western blots with ImageJ (version v1.52r).

For manuscripts utilizing custom algorithms or software that are central to the research but not yet described in published literature, software must be made available to editors and reviewers. We strongly encourage code deposition in a community repository (e.g. GitHub). See the Nature Portfolio guidelines for submitting code & software for further information.

Data

Policy information about availability of data

All manuscripts must include a data availability statement. This statement should provide the following information, where applicable:

- Accession codes, unique identifiers, or web links for publicly available datasets
- A description of any restrictions on data availability
- For clinical datasets or third party data, please ensure that the statement adheres to our policy

All data supporting the findings of this study are available within the paper (and supplementary information files). Source data are provided with this paper.

Field-specific reporting

Please select the one below that is the best fit for your research. If you are not sure, read the appropriate sections before making your selection.

☒ Life sciences ☐ Behavioural & social sciences ☐ Ecological, evolutionary & environmental sciences

For a reference copy of the document with all sections, see nature.com/documents/nr-reporting-summary-flat.pdf

Life sciences study design

All studies must disclose on these points even when the disclosure is negative.

Sample size	N/A
Data exclusions	No data were excluded.
Replication	Replication is stated in the figure legends and/or the Methods section.
Randomization	Placebo effects or biased interpretation of data are not relevant to this study due to the nature of the presented data (northern blots, western blots, Southern blots, images of plant phenotypes, bacteria radial diffusion assay). Quantifications were done with standard software packages, and are unbiased. Therefore, no randomization needed to be performed.
Blinding	Placebo effects or biased interpretation of data are not relevant to this study due to the nature of the presented data (northern blots, western blots, Southern blots, images of plant phenotypes, bacteria radial diffusion assay). Quantifications were done with standard software packages, and are unbiased. Therefore, no blinding was performed.

Reporting for specific materials, systems and methods

We require information from authors about some types of materials, experimental systems and methods used in many studies. Here, indicate whether each material, system or method listed is relevant to your study. If you are not sure if a list item applies to your research, read the appropriate section before selecting a response.

Materials & experimental systems

n/a	Involved in the study	
☐	☒	Antibodies
☒	☐	Eukaryotic cell lines
☒	☐	Palaeontology and archaeology
☒	☐	Animals and other organisms
☒	☐	Human research participants
☒	☐	Clinical data
☒	☐	Dual use research of concern

Methods

n/a	Involved in the study	
☒	☐	ChIP-seq
☒	☐	Flow cytometry
☒	☐	MRI-based neuroimaging

Antibodies

Antibodies used	anti-HA; GenScript THE TM HA-tag Antibody, mAb, mouse, catalogue No: A01244-100 diluted to 0.5 µg/mL<br>anti-mouse antibody HRP-conjugated Sigma A9044; diluted 1:10.000
Validation	"THE™ HA Tag Antibody, mAb, Mouse (GenScript, A01244) is a high-affinity monoclonal antibody that can be used to detect HA-tagged proteins." (https://www.genscript.com/antibody/A01244-THE_HA_Tag_Antibody_mAb_Mouse.html)<br><br>"Anti-Mouse IgG (whole molecule)–Peroxidase antibody produced in rabbit"<br>https://www.sigmaaldrich.com/NL/en/product/sigma/a9044?<br>gclid=Cj0KCQjw94WZBhDtARIsAKxWG-9NZ9d8pixZ-5dpujFBlKMOGiVhBi9b8S2xA0HeoEii__NIUI57Rb4aAkbfEALw_wcB
